# Design and evaluation of additive manufactured highly efficient inclined-wing type continuous mixer

**DOI:** 10.1038/s41598-022-23809-2

**Published:** 2022-11-14

**Authors:** Seoung-Ho Baek, Jung-Ho Yang, Cheol-Woo Ha, Patrick Y. Shim, Son Yong, Sang-Hu Park

**Affiliations:** 1 Research Institute of Machinery and Technology, Pusan National University, Geumjeong-Gu, Busan, 46241 Korea; 2 Advanced Joining and Additive Manufacturing R&D Department, Korea Institute of Industrial Technology, Siheung, 15014 Korea; 3 G&I Solution Co. Ltd, Siheung, 14922 Korea; 4 School of Mechanical Engineering, Pusan National University, Busan, 46241 Korea

**Keywords:** Chemical engineering, Mechanical engineering, Chemistry, Engineering, Materials science

## Abstract

We develop a novel milli-scale mixer (tilted-wings mixing unit, TWM unit) based on the design for additive manufacturing (DfAM). The proposed tilted-wings mixer has basically designed to have three separate wings that split and combine fluids in order to mix together effectively. Its structure is simple for easy fabrication: two major design parameters of angle among three wings and connecting angle between tilted-unit, which are optimized using the computational fluid dynamics (CFD) analysis. From the CFD analysis, we obtain the best-combined mixing module from analyses of various combinations of TWM units for a highly effective mixing ratio. The mixing ratio of three combined units reaches near 100%, which is validated by the experiment and analysis. We believe that the proposed milli-scale mixer can be utilized in diverse chemical continuous mixers and reactors for minimizing of use of chemicals that can pollute the environment.

## Introduction

Mixing fluids is an important process in chemical engineering^1,2^, food engineering^3^, electronics, mining^4^, and others. Until now, lots of studies have been conducted to improve the efficiency of the mixing ratio with diverse mixer designs^3,4^. As the advancement of various industrial fields and environmental issues are raised, the mixing process of chemicals requires high performance as well as low pollution and safety^2–7^. For example, PPO (polyphenylene oxide), is one of the key materials for fifth-generation (5G) communication antennas with good electrical performance, low dielectric loss, and small change in dielectric performance with a wide range of frequencies. However, when PPO is mixed using a batch-type mixer, which is generally adopted in chemical plants due to low production cost, there is a risk of explosion, and it is difficult to obtain a high yield of mixture^8^. To solve the limitations of the batch-type mixers, many research works have been reported on continuous mixers owing to high mixing performance, safety, ease of control, scalability, and low pollutant generation compared to the characteristics of the batch-type mixers^9,10^.

A continuous mixer has some process conditions such as Reynolds number (*Re*), fluid type, and amount of fluid flow. Based on the mixing conditions, diverse continuous mixers have been proposed; chaotic mixer^11^, triply periodic minimal surface (TPMS) mixer^12^, horizontal and vertical weaving (HVW) mixer^13^, and Kenics^14^. Especially, lattice-structure based mixer (LSM) has received a lot of attention due to its high mixing efficiency compared to its length. It usually consists of complex intersecting bars or rods (normally ten or more), and the fluid mixes together as it passes through a lattice structure. Therefore, the designed shape and structure of the LSM affect the mixing performance. The conceptual design of the LSM was firstly proposed by Sulzer in the 1960s, where several bars inside the mixer perform Baker’s split and recombination to perform the mixing of fluids^15^. The LSM can be designed to have a wide range of *Re* from tens to thousands of fluid flows by change of the number and dimension of bars to control the mixing ratio.

Since the first development of the LSM, increasing the mixing ratio and broadening the scope of application has been the main focus of many researchers. Arimond et al. performed a mixing analysis in the field of passive mixers using a Kenics-type mixer^16^, and Fradette et al. conducted a flow analysis for a lattice-based mixer^17^. Pianko-Oprych et al. performed a mixing analysis for two-phase flow and showed the effect of a mixer structure using computational fluid dynamics (CFD)^18^, and Li et al. studied the flow analysis of non-Newtonian liquids to broaden the applications of the LSM^19,20^. Rauline et al. compared the performance of several mixers using CFD analysis^21^, and Zalc et al. elucidated the principle of mixing in the LSM by velocity distribution^22^. Heniche et al.^23^ and Liu et al.^24^ studied the mixing ratio of the LSM according to the shape of a unit structure. Ghanem et al. summarized previous studies and compiled the shape characteristics, mixing principles, and application fields of the LSM^25^. Hirschberg et al. performed a shape change to reduce the pressure build-up of the LSM^26^, and Shahbazi et al. attempted to optimize the shape of the LSMs using a genetic algorithm^27^.

However, despite the high performance of the LSM, there are many small-scale rods intersecting inside the LSM, making it difficult to fabricate it^23,24^. To solve the manufacturing issue, we utilize the additive manufacturing (AM) process to make a highly performance mixer, and CFD analysis is used to optimize design parameters in liquid-to-liquid mixing. With the recent advances of additive manufacturing (AM) process technology^28–32^, many researchers focusing on AM process in a static mixer. However, many researchers designed simple shapes like a channel-combined mixer unit with Y-shape or split-recombine channel^32,33^. Also, the widely utilized LSM-type mixer designed considering AM process is not popular^34,35^. Therefore, in this work, we newly design the LSM with a simpler shape and higher performance in the mixing of the same viscous fluid using the mixer for a commercial small tube (6.35 mm). It is basically a unit structure consisting of three inclined wings for effective splitting and combining.

In order to validate the proposed mixer, we have conducted experiments and compared the experimental and analysis results. For visualization, a fluid (the type of paint) with relatively high viscosity (3000 mPa s) was used in the experiment, but it is also in the laminar flow region. In this work, the whole processes were described from concept design of the mixer to design optimization, DfAM process, and test results using the fabricated one. The contents of this paper are as follows; “Design of tilted-wing continuous mixer (TWM)”. Concept design and validation of mixing unit considering AM process; “Optimization of the TWM-FWM module”. Design variables selection and optimization of suggested mixing module; “Experiments and discussion”. Specimen fabrication using AM and validation CFD results.

## Design of tilted-wing continuous mixer (TWM)

### Analysis model and conditions

In this study, the mixing performance of the mixer was evaluated using CFD. The multiple physical phenomena such as laminar flow, wall flow, turbulence, and mixing should be considered for the CFD model of the mixing process. Especially, to evaluate the mixing ratio of a mixer, it is necessary to trace the volume fraction of each fluid. Flow-3D (Flow Science Inc., USA), which specializes in the volume of fraction (VOF) analysis^32,33^, was therefore used to perform mixing analyses.

The analysis was performed based on Eq. (1) which is the equations for continuative equation of incompressible fluid; and Eq. (2) which means momentum conservation equation of incompressible fluid considering turbulence fluids; and Eq. (3), which describes two-phase flow; and Eq. (4), which expresses the VOF technique. In these equations, $\overrightarrow{\mathrm{v}}$ is the average velocity, $\mathrm{P}$ is the pressure, $\uprho$ is the density of the fluid, $\mathrm{g}$ is the gravitational acceleration, $\upmu$ is the viscous coefficient, $\mathrm{f}$ is the volume fraction, $\overrightarrow{{\mathrm{v}}_{1}}$ is the velocity of fluid 1, $\overrightarrow{{\mathrm{v}}_{2}}$ is the velocity of fluid 2, and $\overline{\overrightarrow{\mathrm{v}}\overrightarrow{\mathrm{v}}}$ is the turbulent stress. $\mathrm{f}$ always has a value between 0 and 1. $\mathrm{f}=0$ means that no fluid exists in an area, and $\mathrm{f}=1$ indicates that a fluid exists in an entire area. Despite of the Re of the mixer targeted in this paper is 625, which is in the range of laminar flow, local turbulent flow can occur due to internal structure. Therefore, $\mathrm{k}-\upomega$ turbulence model was adopted^36,37^.

1 $$\nabla \cdot \overrightarrow{\mathrm{v}}=0$$

2 $$\uprho (\frac{\partial \overrightarrow{\mathrm{v}}}{\partial \mathrm{t}}+\overrightarrow{\mathrm{v}}\cdot \nabla \overrightarrow{\mathrm{v}})=-\nabla \mathrm{P}+\uprho \overrightarrow{\mathrm{g}}+\upmu {\nabla }^{2}\overrightarrow{\mathrm{v}}+\overline{\overrightarrow{\mathrm{v}}\overrightarrow{\mathrm{v}}}$$

3 $$\overrightarrow{\mathrm{v}}=\mathrm{f}\overrightarrow{{\mathrm{v}}_{1}}+(1-\mathrm{f})\overrightarrow{{\mathrm{v}}_{2}}$$

4 $$\frac{\partial \mathrm{f}}{\partial \mathrm{t}}+\nabla \cdot \left(\overrightarrow{\mathrm{v}}\mathrm{f}\right)=0$$

**Figure 1:**
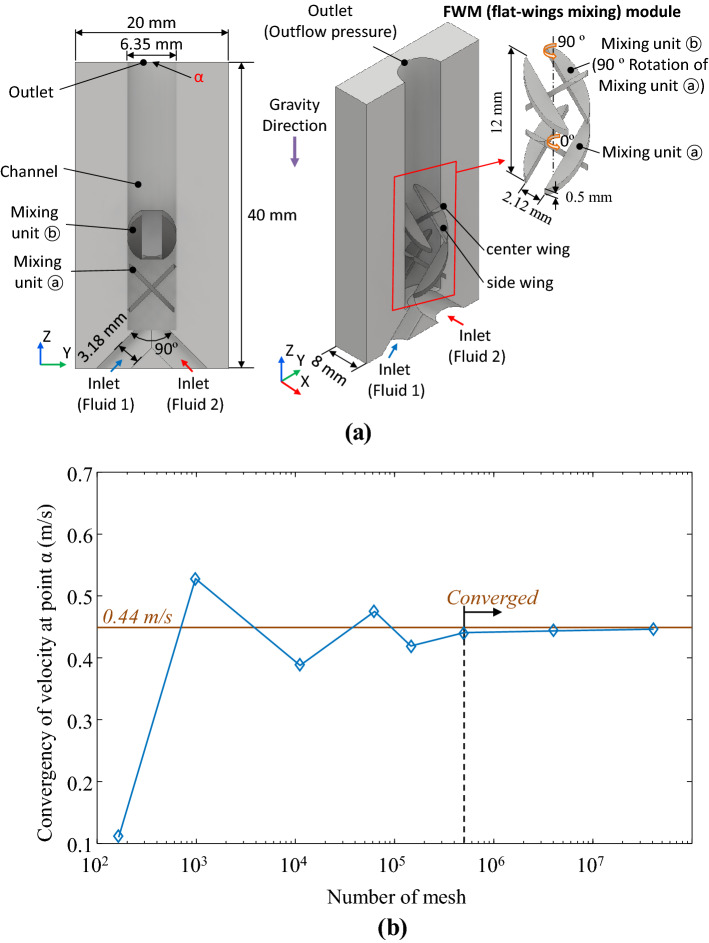
Analysis condition of mixing. (**a**) Boundary conditions and geometries of the mixing analysis. The basic analysis model consisted of two inlets (each diameter of 3.18 mm) and 1 outlet (diameter of 6.25 mm) with a flat-wings mixing module (FWM module) placed and (**b**) results of mesh convergency according to various mesh condition at point α shown in (**a**); convergence condition is the mesh number of more than 5 × 10^5^.

As shown in Fig. 1a, The channel geometry of the analysis model was Y-shaped with two inlets and one outlet. And the diameters of the inlet parts were 1/8 in. (3.18 mm), and outlet part was 1/4 in. (6.35 mm), and the angle between the two inlets was 90°. In this channel, a basic model having three flat wings mixer (FWM) module was placed at the channel. The thickness and total length of the inserted mixing structure are 0.5 mm, and 12 mm each, and the width of the three flat wings is equally 1/12 in. (2.12 mm) is in the scale of milli-scale. As depicted in the FWM mixing module of Fig. 1a, arranged two units (mixing units ⓐ, ⓑ) are placed in pairs, and each unit is rotated at an angle of 90°. The rotation between each unit serves to enhance the mixing performance^27,32^.

The boundary conditions for the mixed analysis were shown in Fig. 1a, the outflow pressure (which means continuative boundary in FLOW-3D) at the outlet was selected, and neglected friction of the wall. The analysis model has a z-direction gravitational acceleration. Considering the size of the mixing model, the analysis area was set to 8 × 20 × 40 mm (x × y × z-direction). The wall was assumed to be in non-slip condition, and the effects of heat transfer and surface roughness were ignored. The size of the analysis area in the z-direction was appropriately changed according to the number of mixing units. To verify the mesh quality, an analysis was conducted under various mesh conditions based on the above analysis conditions. It was assumed that both mixed fluids (Fluid 1, 2) had the material properties of water (viscosity and density of 1000 kg/m^3^ and 1 mPa s, respectively, therefore has excellent compatibility) and surface tension is ignored. And the flow rate of each inlet was 0.1 L/min.

Therefore, the flow of channel a Re of 668 which is a region of laminar flow. As shown in Fig. 1b, it was confirmed that at the same point α at Fig. 1a (outlet) converged at a mesh number of 5 × 10^5^ or more in steady-state. Therefore, a mesh size of 5 × 10^5^ was selected to take the analysis time into account.

### Mixing mechanism

**Figure 2:**
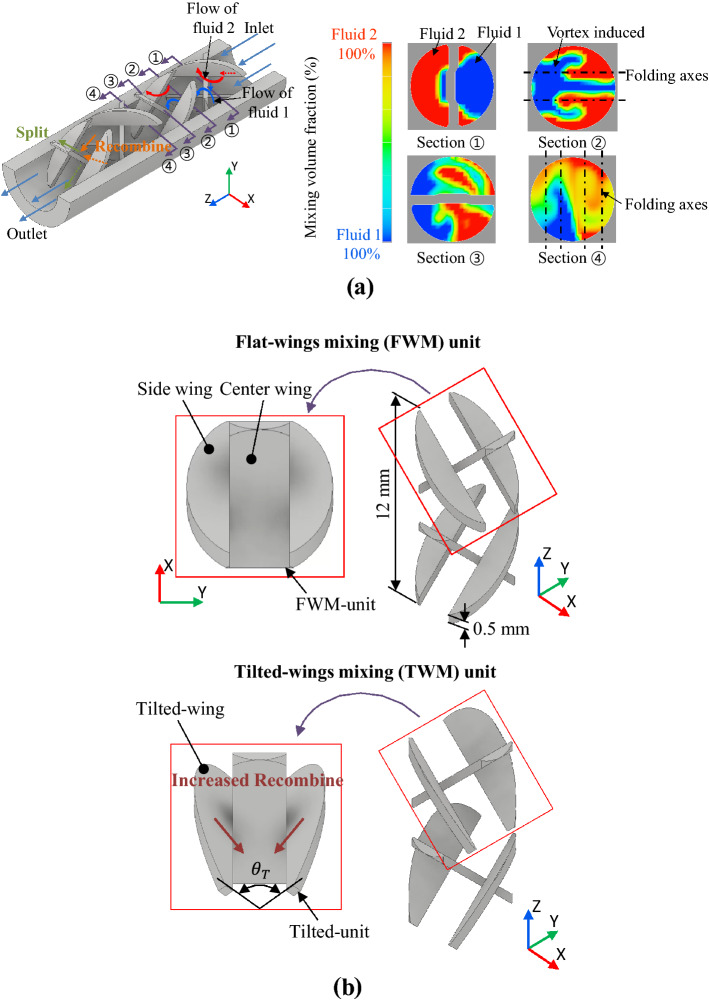
Mixing mechanism and shape of mixing unit. (**a**) Mixing mechanism for FWM unit and cross-section; the color contour of the section shows a mixing volume fraction. Baker's folding process (split and recombination) is performed due to the turbulence caused by wings of mixing unit, and (**b**) cocrison of mixing performance of FWM unit and tilted wing unit (TWM unit), which has ${\theta }_{T}$ (degree between tilted-wings) for increasing recombination function to enhance mixing performance.

A general milli-scale continuous passive mixer achieves mixing by folding and stretching fluids by splitting and recombination them. This is known as the Baker folding process^38^. Likewise, due to the geometry of the FWM, split and recombination of the flow occur to achieve mixing (Fig. 2a). In two-fluid mixing, with fluid passing the FWM, the mixing pattern according to CFD in the cross-section of the channel is shown at ①, ②, ③, and ④ in Fig. 2a^26,27,32,34^. The color of the section means mixing volume fraction, which is the ratio of the volume occupied by each fluid and the volume of the unit mesh.

During parallel two-fluid passing of the first FWM unit, section ① shows fluid-1 passes the center wing and section ② displays that fluid-1 flows from the center wing parts to the side wing section and generates a vortex. Contrary to fluid-1 and fluid-2 flows in the opposite direction (from the side wing to the center wing) as depicted in LSM^26,27,32,39^. This is two rotating vortices cause split fluid and create folding based on two folding axes in Fig. 2a and recombine during rotation. Likewise, while the mixture passes through the second FWM, the split and recombination of the mixture occur by generating four folding axes^17,25,27,32^.

**Figure 3:**
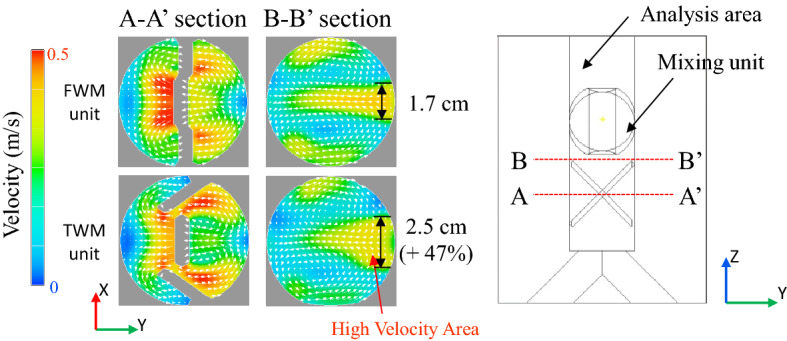
Velocity distribution of the FWM unit and the TWM unit; by comparing each section ② of the FWM unit and the TWM unit, a high-velocity area appears in the middle part of the TWM unit (47% larger than that of the FWM unit). It means the TWM unit has a higher recombination function than the FWM unit.

Considering the mixing mechanism described above, the mixing performance of the FWM unit can be improved by rotating the side wings, as shown in the tilted wing mixer (TWM) unit in Fig. 2b. In this work, a mixer with tilted side wings is called a TWM to distinguish from FWM, the positioning angle between two tilted-wings is *θ*_*T*_. The tilted-wing induces a flow in the transverse direction and strengthens the recombination function. To confirm the mixing mechanism of the TWM unit, the flow patterns of the basic types of FWM units and TWM units were compared. Figure 3 shows the velocity distribution at the FWM unit and TWM unit sections. In sections A–A′, for the TWM unit, a higher velocity transverse directional flow occurs in front of the tilted-wing. As shown in Fig. 3, this induces about 47% widening of the high-velocity area in the TWM unit compared with the FWM unit at the center of the channel in the section-B′. And this large central high-velocity area means that the TWM unit can reinforce the mixing of fluids by increasing the recombination of fluids through transverse directional flow.

### Combination of the TWM and FWM unit

To verify the effect of the TWM on the design of the mixing module, the mixing performance of it should be evaluated quantitatively. Especially, as mentioned above, the FWM module paired two FWM units which have 90° between each unit. Therefore, mixing the performance of the paired modules of the FWM and TWM units are required.

The mixing performance can be evaluated by quantification using the standard deviation of the mixing volume fraction (${\upsigma }_{\mathrm{VF}}$) in Eq. (5)^38,39^. The volume fraction can be referred to as the concentration of each fluid. Therefore, ${\upsigma }_{\mathrm{VF}}$ means standard deviation of concentration, which is directly related to mixing performance. This means that ${\upsigma }_{\mathrm{VF}}$ can be used as an index to quantify mixing performance. The process of calculating ${\upsigma }_{\mathrm{VF}}$ is as follows. In Eq. (5), ${\mathrm{N}}_{\mathrm{t}}$ is the number of measurement points (nodes), ${\mathrm{C}}_{\mathrm{i}}$ is the volume fraction of Fluid 1 at the i-th point, and ${\mathrm{C}}_{\mathrm{mean}}$ is the average of the volume fraction of all points. The closer ${\upsigma }_{\mathrm{VF}}$ is to 0, the better the mixing performance is because each fluid is present in a similar proportion to the unit volume.

5 $${\upsigma }_{\mathrm{VF}} = \sqrt{\frac{1}{{\mathrm{N}}_{\mathrm{t}}}\sum_{\mathrm{i}=1}^{{\mathrm{N}}_{\mathrm{t}}}(\frac{{\mathrm{C}}_{\mathrm{i}}-{\mathrm{C}}_{\mathrm{mean}}}{{\mathrm{C}}_{\mathrm{mean}}}{)}^{2}}$$

Considering the FWM unit and TWM unit, there are four combinations: FWM-TWM (combined module with the FTM unit at the front and TWM unit at the back), TWM-FWM, FWM-FWM, and TWM-TWM. The ${\upsigma }_{\mathrm{VF}}$ of each combination at the outlet part is listed in Table 1. Based on Table 1, two observations can be made. First, when the TWM unit is placed in front, the mixing ratio increases (compare section⑤ of FWM-FWM and TWM-FWM), and when TWM is placed at the back, the mixing ratio decreases (compare Section⑤ of FWM-FWM and FWM-TWM). This is because the TWM unit enhances the recombination of fluids rather than the splitting of fluids. Second, the mixing ratio of the TWM unit decreases immediately after mixing, but it improves at a certain distance away from the mixer (compare section④ of FWM-FWM, TWM-TWM, and section⑤ of FWM-FWM, TWM-TWM). This is evidence that the flow in the transverse direction occurs because of the TWM unit.

**Table 1:**
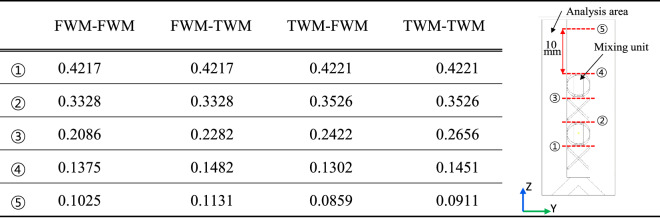
Mixing performance (${\sigma }_{VF}$, standard deviation of mixing volume fraction) according to the combination of FWM and TWM units.

FWM-TWM means the combination of TWM unit at the front and FWM unit at the back.

**Figure 4:**
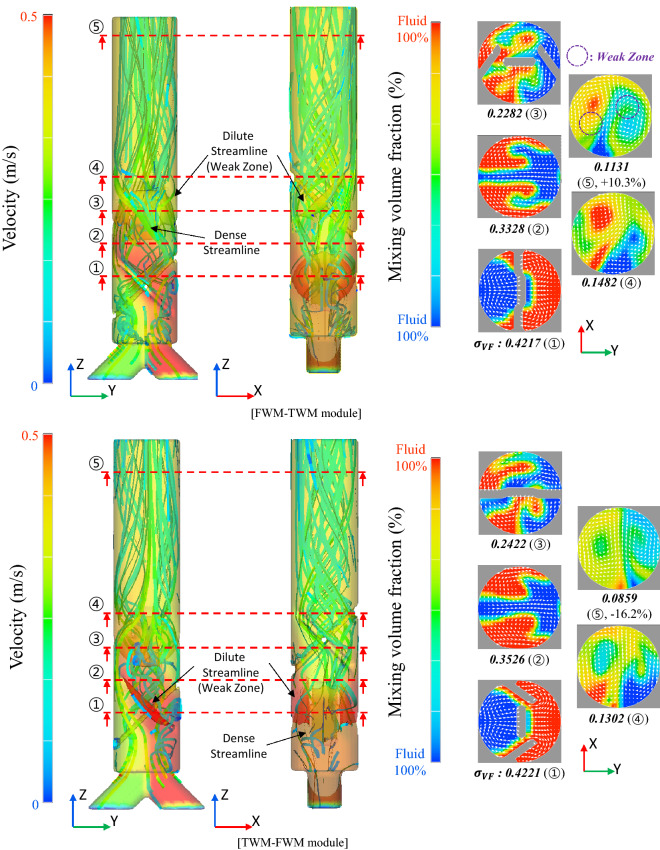
Streamlines and mixing performance of FWM-TWM (combination of with FWM unit at the front and TWM unit at the back) module and TWM-FWM module; transverse direction flow occurs in the TWM unit. Therefore, by placing the TWM unit at the rear, the mixing performance increases by 16% in the mixer with the TWM-FWM module.

To verify the mechanism and design of the mixing structure, the mixing patterns of FWM-TWM and TWM-FWM were compared, as shown in Fig. 4. In Fig. 4, the color of the transparent part where the streamline appears indicates the flow velocity, and the red area in the cross-sections of Fig. 4 indicates the area fully occupied by fluid 1, and the blue area indicates the area fully occupied by fluid 2. If the red and blue areas are mixed, the yellow or green area increases. The values stated below for each cross-section represent the evaluated ${\upsigma }_{\mathrm{VF}}$ for each cross-section. As mentioned above, compared to the FWM unit, the TWM unit increases the flow of the transverse directional and the high-velocity area, which improves the mixing performance. However, as shown in Fig. 4, right behind the TWM unit, a “weak-zone” is formed. In this area, mixing is not performed well because momentum is not transmitted. This weak-zone can also be confirmed by the fact that the streamline hardly appears immediately after the TWM unit (“Dilute Streamline” in Fig. 4). Therefore, the FWM-TWM module, which is a TWM unit, is placed at the rear, and the mixing performance is lower than that in the FWM-FWM module. However, the TWM-FWM module has a better mixing ratio than the FWM-FWM module, which means that the FTM unit placed at the rear removes the weak-zone of the TWM unit. In other words, although the TWM unit increases the mixing performance in a certain area, there is a limit to the improvement because a weak-zone is partially generated behind the TWM unit. However, the FWM unit behind the TWM unit removes the weak-zone. This is why the best mixing performance is achieved with the TWM-FWM module. Accordingly, the TWM-FWM module was selected as the optimum combination of the mixing module. The shape of the TWM-FWM module is optimized in “Optimization of the TWM-FWM module”.

## Optimization of the TWM-FWM module

### Angle *θ*_*T*_ of TWM-FWM module

The design variables for the TWM-FWM module including the TWM were as follows: firstly, the angle between the wing parts of the TWM unit (*θ*_*T*_), secondly, the width ratio of the side wing (α), and half-width of the center wing (β) of the FWM unit and TWM unit (width ratio). Then, the mixing ratio according to the number of modules was evaluated for the optimized TWM-FWM module.

**Figure 5:**
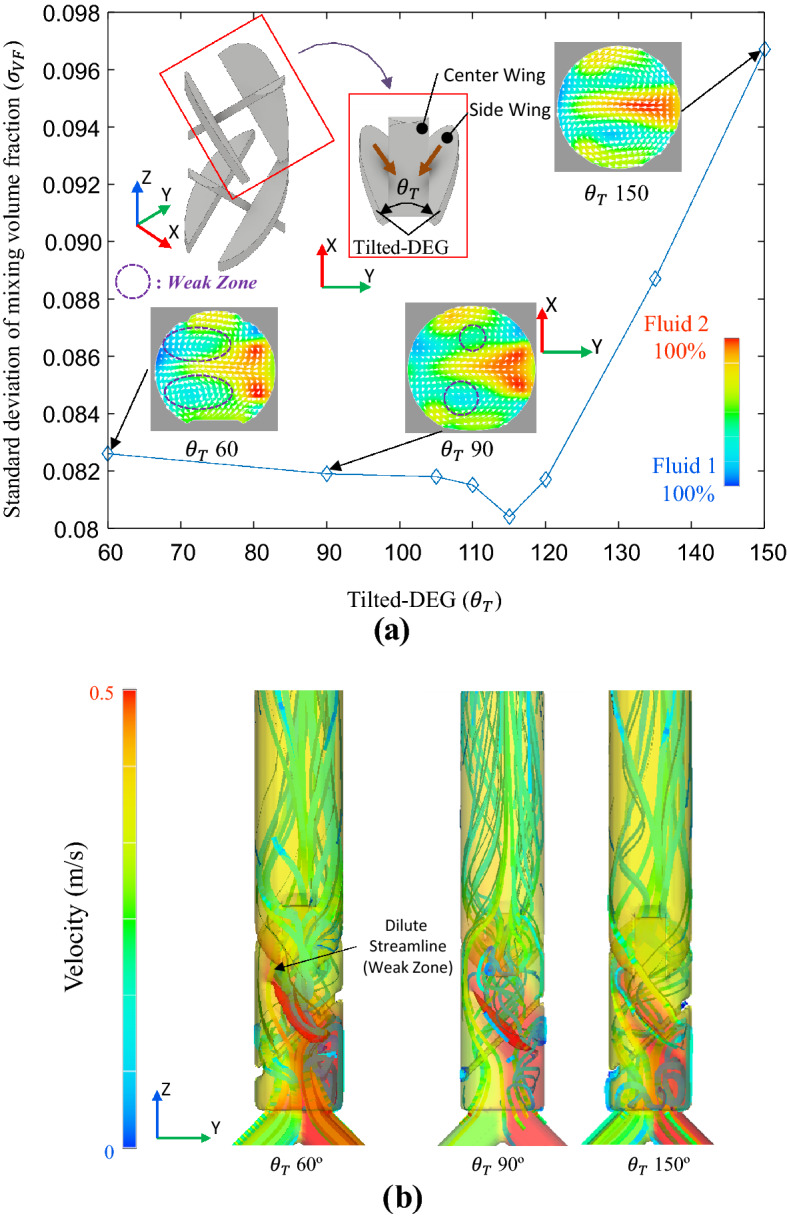
(**a**) Mixing performance according to *θ*_*T*_; As *θ*_*T*_ is away from 115°, a small area of high-velocity distribution occurs, and (**b**) streamline according to variation of *θ*_*T*_; as *θ*_*T*_ decrease, weak-zone is increased (at *θ*_*T*_ = 60°).

As explained in “Design of tilted-wing continuous mixer (TWM)”, the *θ*_*T*_ leads to the generation of momentum in the transverse direction, but a weak-zone is also generated. It is, therefore, necessary to simultaneously generate an appropriate weak-zone that can sufficiently distribute flow to the FWM unit and high momentum in the transverse direction through an appropriate *θ*_*T*_ for a high mixing ratio to be realized. As a result, an analysis of the TWM-FWM module with various *θ*_*T*_ revealed that ${\upsigma }_{\mathrm{VF}}$ was 0.0804 for *θ*_*T*_ at approximately 115°, showing the minimum mixing ratio (see Fig. 5a). This is about 21.6% higher than the 0.1025 of the simple FWM-FWM. In Fig. 5a, when *θ*_*T*_ is larger than 115°, momentum in the transverse direction is hardly generated. Therefore, the region showing a high-velocity distribution decrease at *θ*_*T*_ of 150°. Accordingly, the effect of improving the mixing ratio of the TWM unit is reduced, and ${\upsigma }_{\mathrm{VF}}$ is reduced to 0.0967 at *θ*_*T*_ of 150°. This is an improvement of 5% compared to the mixing ratio FWM-FWM, but it is 16%p lower than the optimal result.

In the contrast, if *θ*_*T*_ is smaller than 115°, the weak-zone increases excessively that cannot be equalized through the FWM unit, and the mixing ratio decreases. Therefore, in Fig. 5b, the streamline at the point where *θ*_*T*_ is 60°, streamline hardly occurs (dilute streamline) owing to the small *θ*_*T*_ of the TWM unit. In the case of mixing ratio *θ*_*T*_ at 60° is 0.0826, which is 19% higher than FWM-FWM, but it is 2.6% p lower than the optimal result. In addition, the mixing ratio responds more sensitively to changes in *θ*_*T*_ when *θ*_*T*_ is large than when *θ*_*T*_ is low compared to 115°. This suggests that the decrease in mixing performance in the case of *θ*_*T*_ smaller than the optimal value is from the failure of the remove the weak-zone through the FWM unit.

### Width ratio α/β of TWM-FWM module

**Figure 6:**
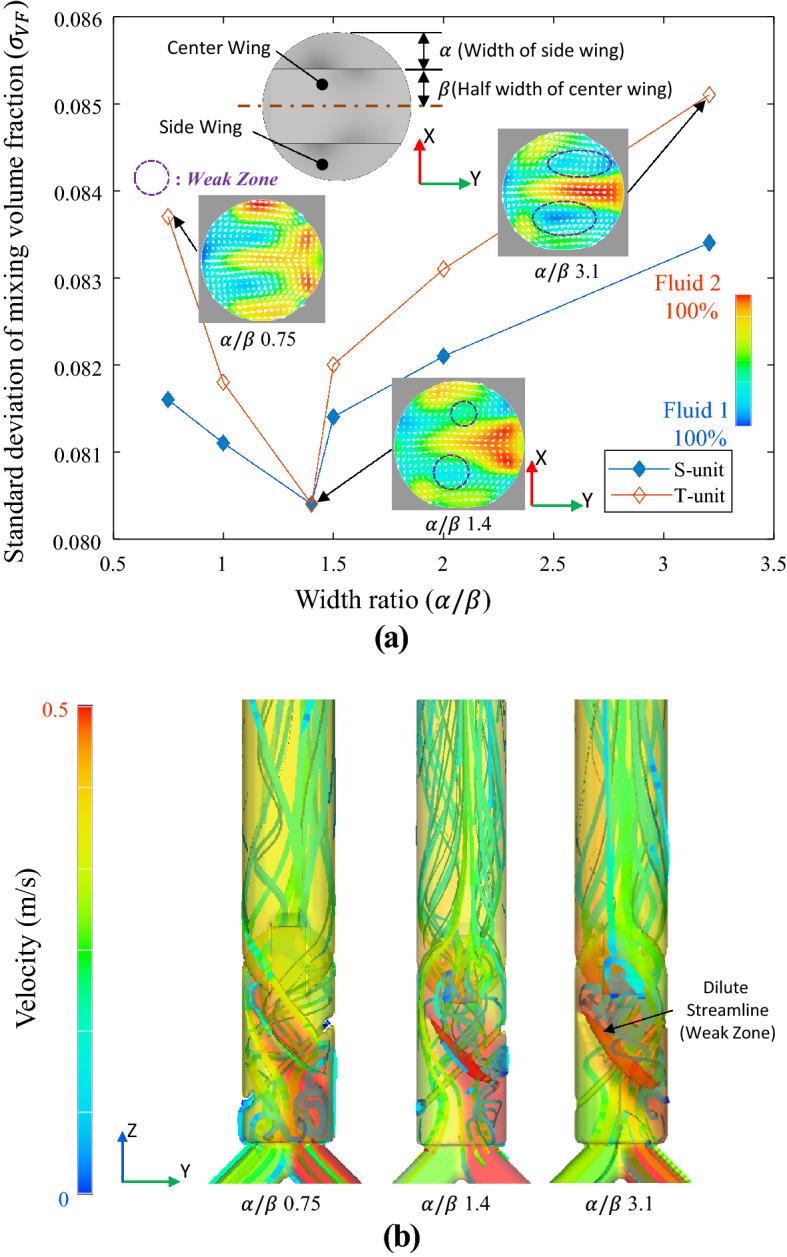
Mixing performance according to the ratio of α/β; as the α/β is par from 1.4, a small area with a high-velocity distribution is generated; (**b**) streamlines according to the ratio of α/β. As the α/β increases, the size of weak-zone also increases (at α/β = 3.1).

The α/β is the width ratio of the side wing (α) to the half-width of the middle wing (β) based on the cross-section projected on the flow path. Because α/β affects the mixing ratio, to select an appropriate α/β, the mixing ratio was evaluated according to the α/β as Fig. 6a. In Fig. 6a, TWM unit has *θ*_*T*_ of 115° as designed in “Angle *θ*_*T*_ of TWM-FWM module”. As shown in Fig. 6a, the mixing ratio is the best when α/β of both the FWM unit and TWM unit is 1.4, and the mixing ratio decreases when α/β departs from 1.4. As the area of the wing portion increases (when α/β increases), the flow rate past the center wing decrease. This can also be confirmed by the appearance of a small high-speed region at the high α/β point (α/β of 3.1) in Fig. 6a. Moreover, in the case of the TWM unit, large α/β increase the weak-zone excessively. This can be confirmed in Fig. 6b, where the streamline at α/β is 3.1, and the streamline hardly occurs (dilute streamline) owing to the high α/β.

Therefore, basically decreases in mixing ratio, in this case, is due to reducing the splitting function due to unbalanced flow in the center and the side wing. This is why the TWM unit is more sensitive to α/β than the FWM unit (when the α/β increases by 0.1 (from 1.4 to 1.5), the ${\upsigma }_{\mathrm{VF}}$ of the FWM unit increases by 0.001 (from 0.0804 to 0.0814), while that of the TWM unit increases by 0.0018 more (from 0.0804 to 0.0822)). In the case of small α/β, a similar effect is observed. As the area of the wing portion decreases (when α/β decreases), the flow rate past the side wing decrease, splitting function also decrease. However, a decrease of α/β generates a small weak-zone, a decrease in α/β is less pronounced for mixing ratio than an increase.

### Number of TWM-FWM module

**Figure 7:**
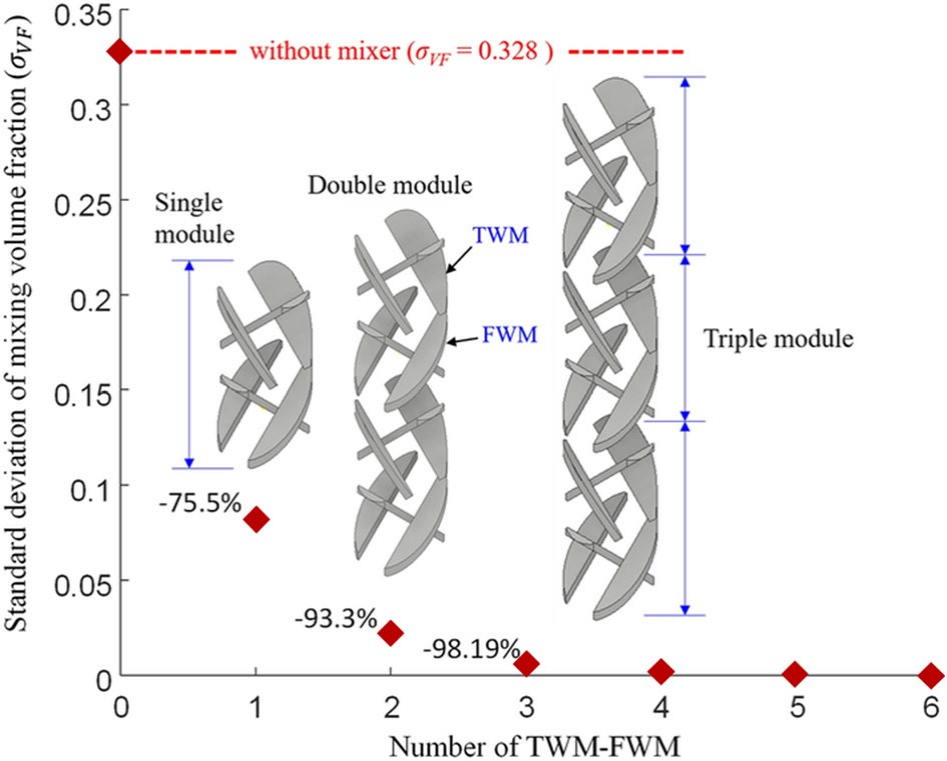
Mixing performance according to the number of optimized TWM-FWM modules; as the number of TWM-FWM modules increases, ${\sigma }_{VF}$ decreases exponentially. When the TWM-FWM module number is three, ${\sigma }_{VF}$ decreases by 98.19%.

Generally, the mixer consists of several arranged mixing modules^25–27,39^. Therefore, the arrangement number of the commercial mixing module is selected in consideration of manufacturability and maintenance. However, because AM is used, unlike for the existing mixer, the number of mixing modules does not affect manufacturability in the TWM-FWM module, as long as the build size of the equipment allows it. However, since it is important to select the appropriate number of mixers in terms of maintenance and repair, the mixing performance according to the number of mixing modules was analyzed in Fig. 7 based on the optimal case in “Optimization of the TWM-FWM module” (*θ*_*T*_ is 115° and the width ratio (α/β) is 1.4).

As shown in Fig. 7, ${\upsigma }_{\mathrm{VF}}$ of the pipe when the mixing module was not installed was 0.328, and when one TWM-FWM module was installed, ${\upsigma }_{\mathrm{VF}}$ was reduced by 75.5%. As the number of TWM-FWM module increased, the ${\upsigma }_{\mathrm{VF}}$ decreased. When four TWM-FWM modules were installed, ${\upsigma }_{\mathrm{VF}}$ was 0.00168, a reduction of 99.5%, and when six TWM-FWM modules were arranged, ${\upsigma }_{\mathrm{VF}}$ was 0.000136, which was a 99.96% reduction. This implies that the mixing ratio according to the number of TWM-FWM modules can be expressed as an exponential function like LSMs^25–27,39^. Therefore, it is possible to estimate the mixing ratio based on the number of mixing modules with one mixing module.

## Experiments and discussion

### Fabrication and experiment

**Figure 8:**
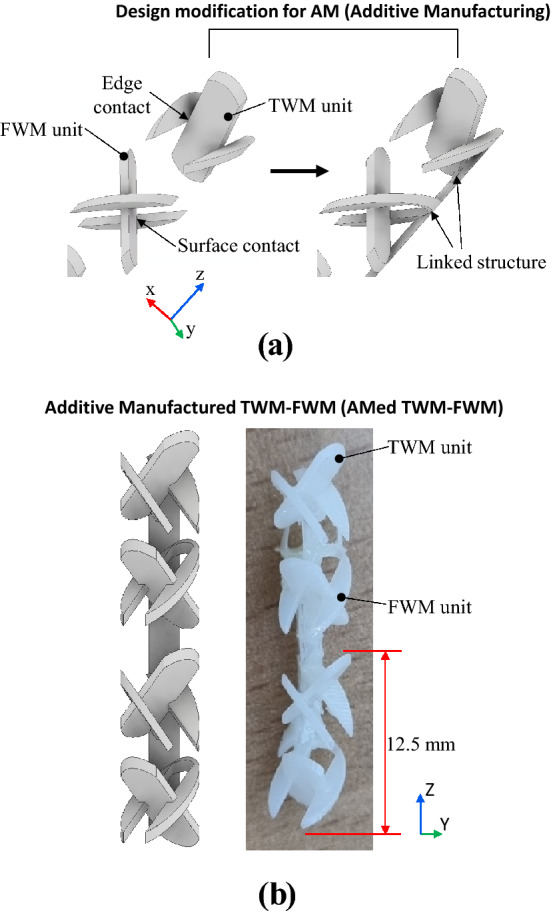
(**a**) Design modification for additive manufacturing. Linking structures between units are added, (**b**) the design geometry and the fabricated specimens of additive manufactured TWM-FWM (AMed TWM-FWM).

To fabricate the proposed TWM-FWM geometry, a shape change was performed so that AM could be used. Two points should be considered for AM^40,41^. First, all parts should be connected by volume, not by surface or edge contacts. Unconnected parts can cause manufacturing problems, such as peeling and collapse during the AM. In addition, to achieve a high-ratio mixer, each mixing unit needs to be connected to maintain a constant angle. This increases the need for all the structures to be connected. Second, supports should be minimized^40,41^. The supports are a structure that is printed together with a part during AM to prevent structural collapse and increase heat dissipation. However, supports negatively affect the product, such as by reducing the surface roughness of the product and impairing aesthetics. Moreover, because the size of the suggested mixing structure is on the milli-scale, there is a risk that the specimen can be damaged during the process of removing the supports. The shape design was, therefore, changed as shown in Fig. 8a. In Fig. 8a, the edge and surface contact parts of each TWM and FWM are connected, and the FWM unit and TWM unit are connected to a linked structure. The additive manufactured TWM-FWM (AMed TWM-FWM) which is modified through this process can be manufactured without supports if it assumes an appropriate position. The AMed TWM-FWM was printed using a selective vat photopolymerization type equipment (Z-rapid, China, SLA300) and an “ABS-like” material. As shown in Fig. 8b, all parts of specimen are well printed and the fabricated thickness was in a range of 0.48–0.51 mm. Compared with their design thickness of 0.5 mm, a dimensional error reaches within about 4%. In addition, the designed length of the TWM-FWM unit was 12 mm, which is the same as the design. Therefore, it can be confirmed that the designed shape and the specimen are printed properly with no significant difference.

### Experimental results

**Figure 9:**
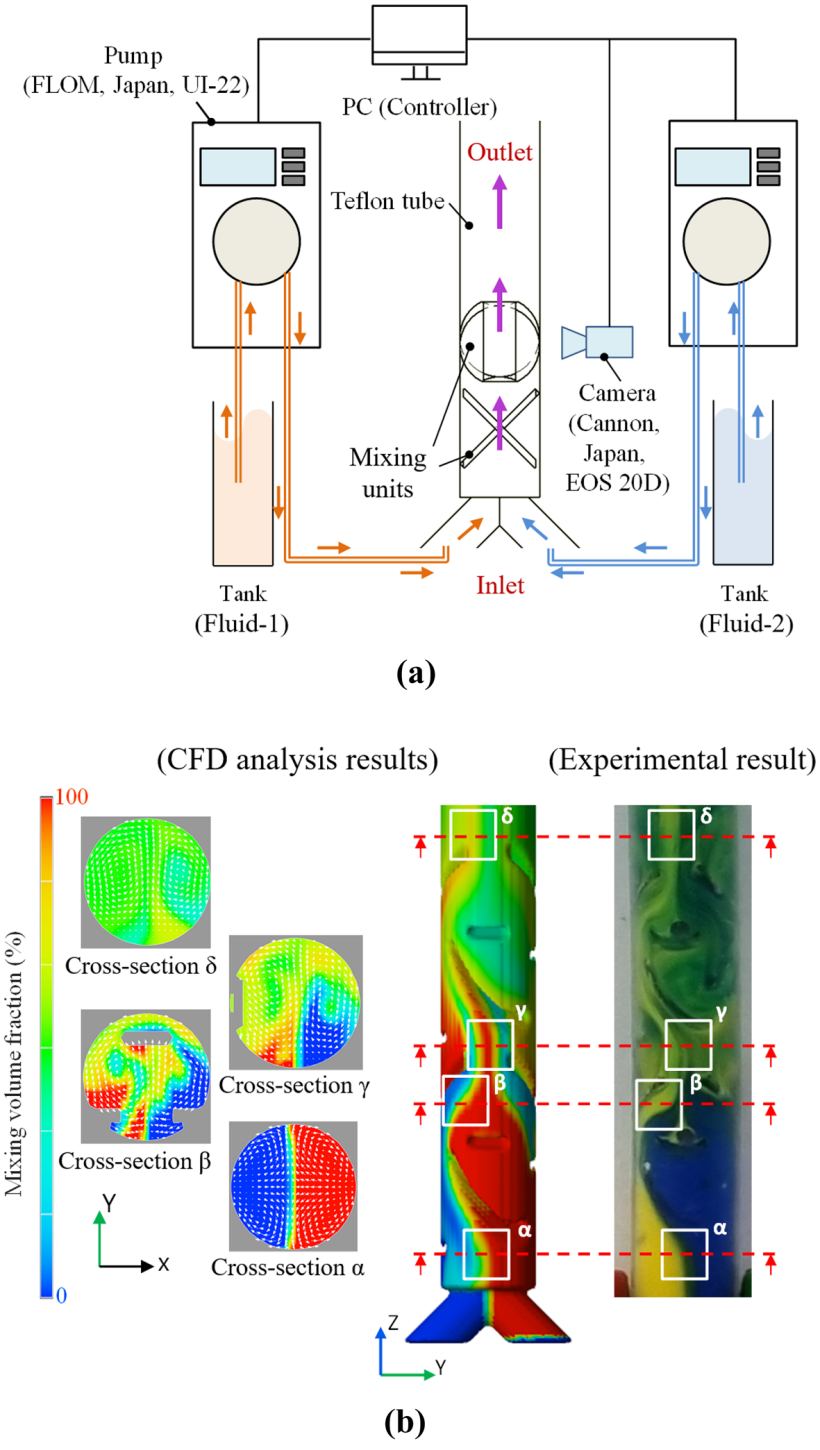
(**a**) Experimental setup for mixing visualization; consisted of two pumps, mixing module in the transparent Teflon pipe, monitoring camera, and controller, and (**b**) comparing of numerical analysis results and experiment; four feature points (α-inlet, β,γ-mixer module, δ-outlet) show similar patterns in numerical analysis and experimental results.

Mixing experiments were conducted using the printed mixer specimens, as shown in Fig. 9a. This experimental system consisted of two intelligent pumps (FLOM, Japan, UI-22, two fluids, a Y-shaped nipple, a camera (Cannon, Japan, EOS 20D), and a controller (PC). The printed specimen was inserted into a transparent Teflon tube so that a streamline of the wall-flow during mixing could be observed. The fluids used in the experiment were oil paints of two colors (blue and yellow) that had a density and viscosity of 1000 kg/m^3^ and 3000 mPa s. The streamlines appearing during the mixing process were visualized and photographed. With intelligent pumps, each fluid flowed at a flow rate of 2 ml/min, and mixing was performed using a mixer installed inside. After a re-analysis was performed considering the experimental conditions, they were verified by comparing them in Fig. 9b.

Figure 9b shows the distribution of the concentration of the fluid. The red parts are areas where the concentration of fluid-1 is 100%, and the blue parts are areas where the concentration of fluid-2 is 100%. In Fig. 9b, at the entrance of the mixer, the part where the flow is bent owing to the TWM unit (α) shows the same pattern in both the numerical analysis and experiment. In the mixing area, where AMed TWM-FWM is inserted in the tube (β, γ), the streamline pattern matches well. In particular, a weak-zone and poor mixing can be observed in the β part. Finally, if we consider the region after the FWM unit where the flow is not mixed, as that region is extended (δ), it can be concluded that the analysis and the experiment are consistent well.

### Discussion

Because it is difficult to implement mixing using turbulence in a small pipe that has several mm diameters, the mixer design in this paper did not consider the flow of the turbulent flow region. However, in general, mixing performance is improved when turbulence occurs, so it is thought that a discussion of the laminar flow region will suffice. In addition, the design strategy presented in this paper is meaningful in that it can be applied without significant changes to the existing mixer design and effectively improves the mixing performance by considering design for additive manufacturing.

In this study, the design was performed based on the most basic FWM structure, a structure with three bars, but this mixer design concept can be applied to the shape of an existing mixer with a larger number of bars by tilting the outside bar. Therefore, by combining the mixer design, it will be possible to design a mixer suitable for various environments such as flow with higher Re. However, when two fluids with significantly different viscosities are mixed, the strength of the flow in the transverse direction may change as the mixing pattern changes. Accordingly, in the case of mixing fluids with different viscosities, optimization of design parameters should be proceeded.

## Conclusion

A simple-shaped high-efficiency milli-scale continuous mixer (named TWM) was developed with an additive manufacturing (AM) based design. The novel mixing unit has three intersecting tilted-wings which can increase the mixing ratio by enhancement of split and recombination. By combination of FWM and TWM unit, we optimized the best mixing module (TWM front – FWM back). The mixing mechanism and performance of the combined mixer were elucidated using CFD analyses and experiments. During mixing in the TWM, there are high and low flow velocity zones, and the distribution of flow velocity is changed in the FWM, so the mixing ratio is rapidly increased through the combination of two units.

By optimizing the TWM-FWM module considering design parameters like the width ratio of a wing and the positioning angle between two units (${\theta }_{T}$), the mixing efficiency increased by about 21% compared to the FWM module only. The suggested TWM-FWM module was fabricated using the AM process and evaluated the performance experimentally. Local mixing states were compared, and the results showed a good agreement between CFD analysis and experiment. Through this work, a simple and effective mixer that can be used in various chemical mixing processes with a small amount of chemicals was developed to reduce material loss and contaminants. Later, optimization of the proposed shape considering the viscosity of various fluids and verification in various flow ranges will be carried out in the future.

## Data Availability

All data generated or analyzed during this study are included in this published article.
